# Correction: Study of reference intervals for free triiodothyronine, free thyroxine, and thyroid-stimulating hormone in an elderly Chinese Han population

**DOI:** 10.1371/journal.pone.0253359

**Published:** 2021-06-10

**Authors:** Jingting Xiong, Shiguo Liu, Kai Hu, Yinxiang Xiong, Pengyun Wang, Liang Xiong

There is an error in affiliation 3 for author Kai Hu. The correct affiliation 3 is: Department of Dermatological, Wuhan Fourth Hospital; Puai Hospital, Tongji Medical College, Huazhong University of Science and Technology.

## References

[1] Xiong J, Liu S, Hu K, Xiong Y, Wang P, Xiong L (2020) Study of reference intervals for free triiodothyronine, free thyroxine, and thyroid-stimulating hormone in an elderly Chinese Han population. PLoS ONE 15(9): e0239579. 10.1371/journal.pone.0239579 34111234 PMC8191890

