# Supplementary material for: Study of reference intervals for free triiodothyronine, free thyroxine, and thyroid-stimulating hormone in an elderly Chinese Han population
Source: PLoS One. 2020 Sep 24;15(9):e0239579. doi: 10.1371/journal.pone.0239579 (PMC7514062; doi:10.1371/journal.pone.0239579)
Supplement: Attachment — Submitted filename: Response to Reviewers.doc [file pone.0239579.s001.doc]

2019-12-01

*PLOS ONE*

Dear Editors and Reviewers:

Thank you for your letter and for the reviewer’s comments concerning our manuscript entitled “Study of reference intervals for main thyroid functions in an elderly Chinese Han population using fully automated chemiluminescence immunoassays” [manuscript number: PONE-D-19-14834]. The comments were all very helpful for revising and improving our manuscript, as well as for providing significant guidance to the researches of our laboratory. We have amended ethics statement statements in the Methods section of the manuscript. We have studied all comments carefully and have made corrections in the manuscript accordingly. We hope that the revised manuscript meets your expectations. The revised portions are marked in red in the manuscript. The main corrections in the paper and the responses to the reviewer’s comments are given below.

Response to the reviewer’s comments:

1. **Comment:** Please provide the ultrasound of the recruited cases, as well as TRAb results. These two parameters are also important to determine whether the included cases are with normal thyroid or not, especially thyroid ultrasound. Visible and palpable goiter is not enough.

**Response:** Thank you for your constructive advice. We agree with you that the above-mentioned two parameters are important to determine whether the included cases had a normal thyroid or not. However, an ultrasound test is not routinely performed for every hospitalized patient and during each health checkup because of financial reasons, among others. Ours was a retrospective study. **We excluded cases that showed obvious goiter upon visual inspection and palpation by a doctor; an ultrasound test was carried out for cases where goiter could not be excluded by palpation. A similar procedure was followed in a previous research^[1]^.**

TRAb tests for antibodies, such as TPOAb, TGAb and TRAb, are used for identifying autoimmune thyroid conditions widely. **However, TRAb tests are not widely used for every hospitalized patient and during each health checkup because of its controversial clinical applicability^[2]^. According to the criteria established by the American Association of Clinical Chemistry (AACC) Academy^[2]^, TSH reference intervals should be established from persons who are not with detectable thyroid autoantibodies, TPOAb or TgAb.** Some other studies that have established RIs for thyroid hormones also used TPOAb and TGAb^[1, 3]^.

2. **Comment:** Please describe why chose LIAISON to test thyroid function. It is better to use more than one method (at least two) to measure thyroid function.

**Response:** Thank you for your kind advice. As is shown in the study of reference intervals for thyroid function, Roche, Abbott, Siemens, and Beckman analyzers have been used for developing reference intervals for thyroid function^[1, 4-6]^; **the LIAISON analyzer also employs fully automated chemiluminescence and performs stably in daily use^[7]^. Reference intervals need to be set separately for different immune detection platforms used in special projects. There are still many hospitals in China that use the LIAISON analyzer to perform thyroid tests for samples daily. It is essential for clinical laboratories to establish reference intervals for the LIAISON analyzer for regular health checkups or evaluation of patients who visit hospitals.**

There are three methodologies (RIA, IMA, and LC-MS/MS) currently being used for the thyroid function test. The use of radioimmunoassay (RIA) is limited due to the resulting radioactive contamination. Liquid chromatography-tandem mass spectrometry (LC-MS/MS) has progressively improved the specificity of the thyroid function test, but its use is also limited because of the requirement of expensive manually operated machinery. **Currently, thyroid testing is performed on serum specimens using fully automated chemiluminescence immunoassays in most clinical laboratories. Until between-method biases are eliminated, it is not feasible to propose universal reference ranges that would apply across methods.**

Thank you very much for considering this work. We look forward to hearing from you soon.

If you have any questions, please feel free to contact us.

Best regards

Sincerely,

Liang Xiong

Department of Laboratory Medicine, Liyuan Hospital, Tongji Medical College, Huazhong University of Science and Technology, Yanhu Road, 39 Wuhan, Hubei, PR China

Phone number: 86-027-86785006

Fax number: 86-027-86793043

xionglianghust@163.com

[1]	Clerico A, Trenti T, Aloe R, Dittadi R, Rizzardi S, Migliardi M et al. A multicenter study for the evaluation of the reference interval for TSH in Italy (ELAS TSH Italian Study)[J]. Clin Chem Lab Med. 2018, 57: 259-267.

[2]	Demers LM, Spencer CA. Laboratory medicine practice guidelines: laboratory support for the diagnosis and monitoring of thyroid disease[J]. Clin Endocrinol (Oxf). 2003, 58: 138-140.

[3]	Zhai X, Zhang L, Chen L, Lian X, Liu C, Shi B et al. An Age-Specific Serum Thyrotropin Reference Range for the Diagnosis of Thyroid Diseases in Older Adults: A Cross-Sectional Survey in China[J]. Thyroid. 2018, 28: 1571-1579

[4]	Ehrenkranz J, Bach PR, Snow G L, Schneider A, Lee JL, Ilstrup S et al. Circadian and Circannual Rhythms in Thyroid Hormones: Determining the TSH and Free T4 Reference Intervals Based Upon Time of Day, Age, and Sex[J]. Thyroid. 2015, 25: 954-961.

[5]	Sriphrapradang C, Pavarangkoon S, Jongjaroenprasert W, Chailurkit L-o, Ongphiphadhanakul B, Aekplakorn W. Reference ranges of serum TSH, FT4 and thyroid autoantibodies in the Thai population: the national health examination survey[J]. Clin Endocrinol (Oxf). 2014, 80: 751-756.

[6]	Amouzegar A, Delshad H, Mehran L, Tohidi M, Khafaji F, Azizi F. Reference limit of thyrotropin (TSH) and free thyroxine (FT4) in thyroperoxidase positive and negative subjects: a population based study[J]. J Endocrinol Invest. 2013, 36: 950-954.

[7]	Bulur O, Atak Z, Ertugrul D T, Beyan E, Gunakan E, Karakaya S et al. Trimester-specific reference intervals of thyroid function tests in Turkish pregnants[J]. Gynecol Endocrinol. 2019.
