# Supplementary material for: Study of reference intervals for free triiodothyronine, free thyroxine, and thyroid-stimulating hormone in an elderly Chinese Han population
Source: PLoS One. 2020 Sep 24;15(9):e0239579. doi: 10.1371/journal.pone.0239579 (PMC7514062; doi:10.1371/journal.pone.0239579)
Supplement: Attachment — Submitted filename: Response to Reviewers.doc [file pone.0239579.s002.doc]

March 19, 2020

Silvia Naitza

Academic Editor

*PLOS ONE*

Dear Editor and Reviewers:

Thank you for your letter and for the reviewers' comments concerning our manuscript entitled "Study of reference intervals for main thyroid functions in an elderly Chinese Han population using fully automated chemiluminescence immunoassays" [manuscript number: PONE-D-19-14834R1].

The comments were extremely helpful for the revision and improvement of our manuscript, as well as for providing significant guidance to our research. We have studied all comments carefully and have made corrections in the manuscript accordingly. We hope that the revised manuscript meets your expectations. The revised sections are marked in red in the manuscript.

The major corrections in the paper and our responses to the reviewer's comments are given below.

Response to the reviewers' comments:

1. **Comment:** Thyroid function - especially TSH and FT3 levels - are affected by a number of factors including drugs and medical illnesses. It isn't clear if the patients that were included were in-patients or out-patients. Older people and those that already have underlying medical conditions are more likely to be admitted to hospital. Thus, changes in thyroid function may reflect their health rather than be due to their age per se. Similarly, a number of drugs such as amiodarone, lithium, metformin and anti-convulsants among others can affect thyroid function. This needs to be clarified.

**Response:** We agree that a number of factors, including drugs and medical illnesses, affect thyroid function. **In this study, all the samples were obtained from in-patients, out-patients, and people who had a checkup done. The excluded ones had thyroid dysfunction, visible or palpable goiter, adrenal insufficiency, renal insufficiency (failure), or other serious systemic diseases; pregnant women or individuals taking drugs that can alter thyroid function test results such as glucocorticoids were also excluded.** This has been revised in the Methods and materials section on page 4.

2. **Comment:** Thyroid function particularly TSH and FT3 levels have a circadian rhythm and can be affected by food. Were the samples obtained at different times of the day and night and in fasting and non-fasting states?

**Response:** Thank you for your kind remind. **Fasting venous blood samples were collected between 8:00 and 10:00 am.** We have added this in the section ‘Subjects and sample collection’ on page 5.

**3. Comment:** Title and abstract: not sure what "main" thyroid functions are?

**Response:** We have revised the mention of main thyroid functions for free triiodothyronine, free thyroxine and thyroid-stimulating hormone in the title and abstract. We changed the title to "Study of reference intervals for free triiodothyronine, free thyroxine, and thyroid-stimulating hormone in an elderly Chinese Han population".

**4. Comment:** Abstract: please explain how the reference intervals were calculated. This is important as this is the main focus of the analysis.

**Response:** The reference intervals were established using the CLSI C28-A3 document: Defining, Establishing, and Verifying Reference Intervals in the Clinical

Laboratory. According to this, the reference ranges of FT3/FT4/TSH were defined by the 2.5^th^ and 97.5^th^ percentile.

**5. Comment:** Introduction (end of first para): several factors affect thyroid function and not just iodine intake and ageing. Please see Jonklaas and Razvi. Lancet Diab Endocrinology 2019 PMID: 30797750

**Response:** It is true that not only iodine intake and ageing but also an inhibitory effect on the hypothalamic-pituitary axis, destructive thyroiditis, or immune-reactivating mechanisms, affect thyroid function. We have made the correction in the Introduction on page 3.

**6. Comment:** Introduction (second para): "Researchers have connected aging with hypothyroidism and believe that hypothyroidism is a manifestation of aging". Please revise this sentence or provide a reference.

**Response:** We revised the sentence "Researchers have recognized that the prevalence of thyroid disease increases with age" and added the following reference: Arch Intern Med. 2000;160:526-534, PMID10695693.

**7. Comment:** Informed consent was not required as this was a retrospective study. Consent has to do with using identifiable information being used with the patient's knowledge and not with the design of the study. Maybe there was no identifiable information available to reserchers and that is why no consent was required?

**Response:** Yes, informed consent was not required as this was a retrospective study. We obtained the necessary information from clinical and demographic information available on the Laboratory Information System (LIS). It can be found in Table 1: guidance on informed consent and ethical approval requirements based on the study type in the field of laboratory medicine. ( https://doi.org/10.11613/BM.2018.030201 PMID: 30429665 )

**8. Comment:** Statistical analysis: this is one of the shortest statistical analysis section I have ever seen. Please provide details of how reference ranges were calculated. And how data are presented.

**Response:** We have made changes as follows: **Statistical analyses were conducted using SPSS statistical software, version 16.0 (Chicago, IL, USA) and GraphPad Prism, version 6.0 (GraphPad Prism Software, San Diego, CA, USA). Kolmogorov-Smirnov tests were used to evaluate the distribution of data; normally distributed data were presented as mean±SD, whereas non-normally distributed data were calculated by the logarithmic transformation. The reference range was defined by the 2.5^th^ and 97.5^th^ percentile (P2.5–P97.5), using SPSS. P-value < 0.05 was considered statistically significant.**

**9. Comment:** Typo on page 10: do you mean rT3?

**Response:** We apologize for the error. We have changed γT3 to rT3.

**10. Comment:** Please include a reference to the recent editorial by Cappola A in JAMA in 2019 The Thyrotropin reference range should be changed in older people. PMID: 31664455

**Response:** We added the sentence "It is necessary to update the thyrotropin reference range for older patients" with the reference on page 4 (Introduction, third paragraph).

Thank you for your consideration. We look forward to hearing from you soon.

If you have any questions, please feel free to contact us.

Sincerely,

Liang Xiong

Department of Laboratory Medicine, Liyuan Hospital, Tongji Medical College, Huazhong University of Science and Technology, Yanhu Road, 39 Wuhan, Hubei, PR China

Phone number: 86-027-86785006

Fax number: 86-027-86793043

Email: xionglianghust@163.com
