# Supplementary material for: Study of reference intervals for free triiodothyronine, free thyroxine, and thyroid-stimulating hormone in an elderly Chinese Han population
Source: PLoS One. 2020 Sep 24;15(9):e0239579. doi: 10.1371/journal.pone.0239579 (PMC7514062; doi:10.1371/journal.pone.0239579)
Supplement: Attachment — Submitted filename: Response to Reviewers.doc [file pone.0239579.s003.doc]

August 23, 2020

Silvia Naitza

Academic Editor

*PLOS ONE*

Dear Editor and Reviewers:

Thank you for your letter and for the reviewers' comments concerning our manuscript entitled "Study of reference intervals for free triiodothyronine, free thyroxine, and thyroid-stimulating hormone in an elderly Chinese Han population" [manuscript number: PONE-D-19-14834R2].

The comments were extremely helpful for the revision and improvement of our manuscript. We have studied all comments carefully and have made corrections in the manuscript accordingly. We hope that the revised manuscript meets your expectations. The revised sections are marked in red in the manuscript.

1.**Comment:** we ask you to further improve the description of your study and describe more thoroughly the details of its retrospective nature, including when the retrospectives searches took place and the date ranges applied when you selected the eligible records.

**Response: We have improve the description in the section ‘Study participants and data collection’on page 4-5 as follows: We conducted a retrospective study focusing on the elderly thyroid function and collected the thyroid function results of 2018 from the Laboratory Information System in 2019. Between Jan. 1, 2018 and Dec. 31, 2018, thyroid function test results of 12,670 individuals were collected from the Liyuan Hospital affiliated to Tongji Medical College of Huazhong University of Science and Technology, Zhongnan Hospital affiliated to Wuhan University Medical College, Hubei Third People’s Hospital, and Wuhan Chinese Medicine Hospital. According to the criteria established by the American Association of Clinical Chemistry (AACC) Academy [17], the total number of individuals without thyroid dysfunction, visible or palpable goiter, treatment with drugs which can alter thyroid function test results such as glucocorticoids, adrenal insufficiency, renal insufficiency (failure) or other serious systemic diseases, or pregnancy was 10,446. Five tests for thyroid function (FT3, FT4, TSH, TPOAb, and TGAb) were conducted and fasting venous blood samples were collected between 8:00‐10:00 am, revealing that 5345 individuals, aged between 19 and 100 years, who met the criteria of being negative for TPOAb and TGAb antibodies.**

**2.Comment:** we kindly ask you to copyedit the manuscript in order to improve the English and gain in clarity. Please also note that we have included a copy of the two reviews on your revised version 2 of the manuscript, so please include the changes suggested by the Referee.

**Response:** We have made some corrections in language expression marked in red in the “Revised Manuscript with Track Changes”. We improved the clarity of picture. And the manuscript include changes suggested by the Referee.

**3.** Reviewer #2 **Comment:** Are all patients asked to attend in a fasting state and between 0800 and 1000 hours or were data collected only for participants who fitted this criteria?

**Response:** We choosed the data collected only for participants who fitted in a fasting state and between 8:00‐10:00 am.

Thank you for your consideration. We look forward to hearing from you soon.

If you have any questions, please feel free to contact us.

Best regards

Sincerely,

Liang Xiong

Department of Laboratory Medicine, Liyuan Hospital, Tongji Medical College, Huazhong University of Science and Technology, Yanhu Road, 39 Wuhan, Hubei, PR China

Phone number: 86-027-86785006

Fax number: 86-027-86793043

xionglianghust@163.com
